# Orange Juice Attenuates Circulating miR-150-5p, miR-25-3p, and miR-451a in Healthy Smokers: A Randomized Crossover Study

**DOI:** 10.3389/fnut.2021.775515

**Published:** 2021-12-24

**Authors:** Mariana S. Dorna, Elizabete M. S. Barbosa, Matheus A. Callegari, Suzana E. Tanni, Fernanda Chiuso-Minicucci, Tainara F. Felix, Ana L. Seneda, Camila R. Correa, Ana A. H. Fernandes, Paula S. Azevedo, Bertha F. Polegato, Marcelo M. Rogero, Sergio A. R. Paiva, Leonardo A. M. Zornoff, Patricia P. Reis, Marcos F. Minicucci

**Affiliations:** ^1^Internal Medicine Department, Botucatu Medical School, São Paulo State University, UNESP, Botucatu, Brazil; ^2^Experimental Research Unit, São Paulo State University, UNESP, Botucatu, Brazil; ^3^Botucatu Medical School, São Paulo State University, UNESP, Botucatu, Brazil; ^4^Chemistry and Biochemistry Department, Institute of Biosciences, São Paulo State University, UNESP, Botucatu, Brazil; ^5^Department of Nutrition, School of Public Health, USP - University of São Paulo, São Paulo, Brazil; ^6^Department of Surgery and Orthopedics, São Paulo State University, UNESP, Botucatu, Brazil

**Keywords:** microRNA, orange juice, smokers, oxidative stress, molecular pathways

## Abstract

**Introduction:** Tobacco smoke is associated with oxidative and inflammatory pathways, increasing the risk of chronic-degenerative diseases. Our goal was to evaluate the effects of acute “Pera” and “Moro” orange juice consumption on inflammatory processes and oxidative stress in microRNA (miRNA) expression in plasma from healthy smokers.

**Methods:** This was a randomized crossover study that included healthy smokers over 18 years old. Blood samples were collected before and 11 h after beverage ingestion. Participants were instructed to drink 400 mL of Pera orange juice (*Citrus sinensis*), Moro orange juice (*Citrus sinensis* L. *Osbeck*), or water. Each subject drank the beverages in a 3-way crossover study design. Inflammatory and oxidative stress biomarkers and circulating miRNA expression profiles were determined. The subjects maintained their usual tobacco exposure during the experiment.

**Results:** We included 18 individuals (12 men and 6 women), with 37.0 ± 12.0 years old. All subjects received the 3 interventions. Increased expression of circulating miRNAs (miR-150-5p, miR-25-3p, and miR-451a) was verified after cigarette smoking, which were attenuated after intake of both types of orange juice. There was no difference regarding serum levels of TNF-α, IL-6, MMP-9, and C-reactive protein. Despite the increased activity of serum superoxide dismutase and glutathione peroxidase after “Pera” or “Moro” orange juice intake, respectively, no changes in lipid hydroperoxide levels were detected.

**Conclusion:** Tobaccos smokers showed increased expression of miR-150-5p, miR-25-3p, and miR-451a was noted, and attenuated by orange juice intake. miRNAs were predicted to regulate 244 target genes with roles in oxidative stress, PI3K-Akt, and MAPK signaling, which are pathways frequently involved in smoking-related cardiovascular diseases and cancer.

## Introduction

Tobacco smoking is the world's leading cause of preventable death, with more than 7 million deaths every year, worldwide (1–3). Tobacco consumption is a known risk factor associated with disease development and mortality by cancer, cardiovascular disease, and respiratory disease (4–6). The high reactive oxygen species (ROS) content found in cigarettes leads to oxidative damage and to further activation of inflammatory pathways related to tobacco-induced adverse effects (7–10). Consumption of fruits and vegetables with anti-inflammatory and antioxidant properties was associated with a reduction in disease development and mortality due to chronic-degenerative conditions, including tobacco-related diseases (11–13). Among such fruits and compounds, orange juice is highlighted because it is one of the most consumed juices in the world (14, 15).

“Pera” orange juice (*Citrus sinensis*) constitutes a rich source of vitamin C, contains various carotenoids, among which the xanthophyll beta-cryptoxanthin in higher concentration, and flavanones such as hesperidin and naringenin (16). Orange juice consumption is considered an efficient means of increasing plasma vitamin C and beta-cryptoxanthin concentration (17, 18). Orange beta-cryptoxanthin is the main precursor of vitamin A in citrus juice, and also has antioxidant properties (18). Recently, blood oranges such as Moro, Torocco and Sanguinello cultivars have been widely studied due to their anthocyanins content. “Moro” orange (*Citrus sinensis* L. *Osbeck*) contains larger amounts of hesperidin than the “Pera,” ranged between 457 to 657 mg/L and around 30 mg/L of anthocyanins, which are not present in Pera oranges (19, 20). Several studies have shown the role of orange juice consumption in preventing heart failure, atherosclerosis, and neurodegenerative diseases (15, 21, 22). These health-promoting effects are mainly due to lowering the blood lipid profile, decrease of plasma interleukin 6 (IL-6) and C-reactive protein levels, and reduction of oxidative stress via the erythroid 2-related factor 2 (Nrf2) pathway (23, 24).

Regarding the orange juices and tobacco exposure interaction, Sánchez-Moreno et al. showed that drinking orange juice (500 mL/d), for 14 days, increased plasma concentration of vitamin C and reduces oxidative stress. Interestingly, in the individuals who reported smoking, concentrations of 8-isoprostane, a biomarker of oxidative stress, were higher compared with non-smokers. In addition, these subjects experienced major reduction in 8-isoprostane after orange juice intake (17). Thus, orange juice intake could have different effects depending on the smoking habit (17, 25).

Acute orange juice consumption has been studied to evaluate other biomarkers and mechanistic pathways that could explain its benefits. Indeed, “omics” technologies, such as genomics, transcriptomics, proteomics, and metabolomics, allow for a comprehensive and global assessment of molecular pathways (26). Based on a metabolomic approach, Moreira et al. showed, in healthy volunteers, that a 2-week orange juice intake affected fatty acids' beta-oxidation through mitochondrial and peroxisomal pathways, leading to an increase of short-chain acylcarnitines and a decrease of medium and long-chain acylcarnitines (27). Orange juice intake was shown to increase pro-myelocytic leukemia protein expression and to downregulate proteins involved in adaptive immune system regulation and cytokine signaling (28).

Despite these studies assessing metabolomic and proteomic profiles after orange juice intake, changes in circulating microRNA (miRNA) expression induced by acute orange juice consumption have not been previously investigated. Importantly, miRNAs are small, non-coding RNA molecules of ~22 nucleotides that act as post-transcriptional regulators of gene expression (29). The study of miRNA expression could help in the understanding of the orange juice health benefits. Likewise, the effects of orange juice intake on inflammatory and oxidative biomarkers of smoking individuals have not been elucidated. Our goal was thus to evaluate the effects of acute “Pera” and “Moro” orange juice consumption in the inflammatory process, oxidative stress, and miRNA expression in the plasma of healthy smokers.

## Materials and Methods

This was a 3-way randomized crossover study approved by the Botucatu Medical School Ethics Committee (85655718.1.0000.5411) and registered at ReBec RBR-97g48p. Signed written informed consent was obtained from all subjects before their inclusion in the study. Sample size was estimated based on a previous study, with orange juice intake after a high-fat and high-carbohydrate meal (28). In this study IL-6 levels reduced more than 65% after the meal consumed with orange juice. Considering this reduction, a power of 90% and type I error of 5% the minimum number to be included was 12 individuals per beverage intake (28).

We evaluated healthy smokers over 18 years old, with current cigarette smoking during hospital outpatient visits, at Botucatu Medical School University Hospital, São Paulo State University, UNESP. Exclusion criteria were the use of antioxidant supplements, use of corticosteroids in the past 3 months, obesity, respiratory diseases, diabetes mellitus, cancer, heart failure, kidney diseases, or hepatic diseases.

Blood samples were collected in the morning of the same day of beverage ingestion. Participants were instructed to drink 400 mL of “Pera” orange juice (*Citrus sinensis*) (“Pera” group), “Moro” orange juice (*Citrus sinensis* L. *Osbeck*) (“Moro” group), or water (Placebo group) and instructed to fast overnight (28, 30). The next morning, 11 h after beverage ingestion, another blood sample was collected. Each subject drank the beverages in a 3-way crossover design. Thus, all the 18 participants received all intervention: water, “Pera” orange juice and “Moro” orange juice. The initial sequence of treatment was determined by simple random sampling. The juices were 100% commercial and pasteurized orange juices, from the same batch, donated by Fundecitrus (“Fundo de Defesa da Citricultura,” Araraquara-SP, Brazil). The washout period was 1 week between treatments. During the follow-up period, subjects were advised to avoid citrus fruits and derivates. In addition, patients continued to smoke during the experimental protocol, including the period between beverage intake and blood sample collection.

### Analysis of Orange Juice Compounds

Carbohydrates, proteins, lipids, ashes, and moisture were determined according to the Association of Official Agricultural Chemists (AOAC) methods (31). Total carbohydrates were calculated according to the following formula: [104 – (g/100 mL moisture + g/100 mL ashes + g/100 mL lipids + g/100 mL proteins)] (31). Total anthocyanin content was measured according to Teixeira et al. (32).

### Serum Beta-Cryptoxanthin Analysis

To determine serum beta-cryptoxanthin concentrations, reverse-phase high-performance liquid chromatography (HPLC) was used as previously described (33). Beta-cryptoxanthin concentration was measured by determining peak areas in the HPLC chromatograms calibrated against known standard quantities. Correction for extraction and handling losses was performed by monitoring recovery of the internal standard.

### Laboratory Data

Total serum levels of albumin, glycemia, creatinine, and urea were measured using the dry chemistry method (Ortho-Clinical Diagnostics VITROS 950®, Johnson & Johnson, Rochester, NY) (34). Total cholesterol (TC), high-density lipoprotein, and triglycerides (TG) were measured by enzymatic colorimetric assay (Ortho-Clinical Diagnostics VITROS 950®, Johnson & Johnson, Rochester, NY). LDL cholesterol was calculated using the Friedewald formula (34). Fasting insulin levels were measured using a chemiluminescence immunoassay. The homeostasis model of assessment of insulin resistance (HOMA-IR) was calculated with the following formula: [fasting glucose (mmol/L) ^*^ fasting insulin (μU/mL)/22.5] (34).

### Serum Inflammatory Biomarkers

TNF-α (DTA00C), IL-6 (D6050), and MMP-9 (DY911) serum levels were assessed using the enzyme-linked immunosorbent assay (ELISA) according to the manufacturer's instructions (R&D System, Inc., Minneapolis, USA) and a microplate reader (Spectra MAX 190, Molecular Devices, Sunny Valley, CA, USA). C-reactive protein (CRP) was measured by turbidimetric immunoassay in an automatic analyzer system (Chemistry Analyzer BS-200, Mindray Medical International Limited, Shenzhen, China) (35).

### Serum Levels of Lipid Hydroperoxide and Antioxidant Enzymes

Serum total protein concentration was determined, and lipid hydroperoxide was measured through hydroperoxide-mediated oxidation of Fe^2+^, as previously described (36). Glutathione peroxidase (GPx), superoxide dismutase (SOD), and catalase (CAT) were assessed as previously specified (36–38). All reagents were from Sigma (St. Louis, MO, USA) (36).

### RNA Extraction

Visual assessment of samples was performed to control for hemolysis. The hemolysis in plasma samples was assessed using a five-level (0, 1+, 2+, 3+, and 4+) scale as reported previously (39). Only samples with a hemolysis level of 0 or 1+ were used for analysis. This technique has been shown to have a sensitivity of 0.25% to detect hemolysis (40).

Total RNA was isolated from 200 μL of plasma (*n* = 5 individuals per group) with the miRNeasy Serum/Plasma Kit (Qiagen, Toronto, ON, Canada), following the manufacturer's protocol (41, 42). This method enables purification of total cell-free RNA from plasma. During extraction, 5 μL of a 200 pM solution of ath-miR-159a exogenous synthetic spike-in control [Integrated DNA Technologies (IDT), Coralville, IA, USA] was added after plasma sample lysis, as previously reported and following the NanoString assay recommendations. Exogenous control was used for data normalization to reduce miRNA expression bias due to differences in RNA yield between different individuals (41, 42). RNA was quantified using Nanodrop 8000 (Thermo Fisher Scientific, Waltham, MA, USA), and samples were stored at −80°C until miRNA expression analysis.

### Circulating Levels of miRNA Expression Analysis

miRNA expression was determined in five patients per group, before and after each intervention. We used the NanoString nCounter® Human miRNA Expression panel v3 (NanoString Technologies, Seattle, WA, USA), which contains color-coded probes for 800 miRNAs with 100% coverage on miRBase (43–45), including miRNAs with clinical relevance (46, 47). From each sample, 200 ng of RNA was subjected to the NanoString nCounter® assay, according to the manufacturer's protocol and as previously reported (48). For data analysis, we used the nSolverTM software (www.nanostring.com/nsolver) for global normalization of miRNA expression data, as well as to determine fold change (FC) and *p*-values for deregulated miRNAs in plasma from smokers, before and after orange juice intake, and compared with controls (water intake).

### Statistical Analyses

Data were expressed as mean ± SD, median (including the lower and upper quartiles), or percentage. A delta between values, before and after the intervention, was calculated (delta = after – before intervention). Statistical comparisons between groups for continuous variables were performed using one-way ANOVA for normally distributed parameters and the Kruskal-Wallis test for non-normally distributed parameters, followed by Tukey's *post-hoc* test. Fisher's or the Chi-square test was used for all categorical data. Data analyses were performed using SigmaPlot software for Windows v12.0 (Systat Software Inc., San Jose, CA, USA). The significance level was set at 5%.

### Computational Data Analyses

Differentially expressed miRNAs were integrated with previously published datasets. This analysis used an existing number of 48.6 million miRNA-target gene inter-actions identified in the microRNA Data Integration Portal (miRDIP) (http://ophid.utoronto.ca/mirDIP/) (49). After identifying predicted miRNA targets, a pathway enrichment analysis with the Enrichr tool (http://amp.pharm.mssm.edu/Enrichr) was used to determine enriched pathways and gene ontologies/biological roles of miRNA target genes (50, 51).

The miRNet 2.0 tool (https://www.mirnet.ca/) was used to generate the interaction network between miRNAs and their target genes (52). A heatmap for significantly enriched pathways regulated by miRNAs and target genes was generated using miRPathDB 2.0 (https://mpd.bioinf.uni-sb.de/) (53).

## Results

A total of 28 healthy smokers were evaluated; however, 4 were excluded due to obesity and 6 due to loss of follow-up. Thus, 18 individuals were included in the study (12 men and 6 women). Mean age was 37.0 ± 12.0 years, and mean BMI was 24.3 ± 3.1 kg/m^2^. Demographic and clinical data are shown in Table 1. It is important to observe that all the 18 subjects received all intervention: water, “Pera” orange juice and “Moro” orange juice, with a washout period of 1 week between interventions.

**Table 1:** Demographic and clinical data of 18 healthy smokers included in the study.

**Variables**	**Values**
Age (years)	37.0 ± 12.0
Male/Female, no.	12/6
BMI (kg/m^2^)	24.3 ± 3.1
Amount of cigarettes (pack year)	9.5 (4.3–16.8)
Fasting glucose (mg/dL)	88.9 ± 8.4
HOMA-IR	2.40 (0.60–8.07)
Albumin (g/dL)	4.28 ± 0.32
Total Cholesterol (mg/dL)	180.1 ± 37.7
HDL-C (mg/dL)	58.6 ± 21.3
LDL-C (mg/dL)	96.1 ± 42.4
Triglyceride (mg/dL)	105.1 ± 61.1
Urea (mg/dL)	28.0 ± 8.2
Creatinine (mg/dL)	0.77 ± 0.14
Hematocrit (%)	43.0 ± 5.1
Hemoglobin (g/dL)	14.2 ± 1.7

*BMC, body mass index; HOMA-IR, Homeostasis Model of Assessment of Insulin Resistance*.

Analysis of orange juice compounds is shown in Table 2. Total anthocyanin content of “Moro” orange juice was 2.83 ± 0.02 mg/mL.

**Table 2:** Orange juices macronutrients compounds.

**Compounds**	**Pera orange juice**	**Moro orange juice**
Moisture (g/100 ml)	91.36 ± 0.01	92.20 ± 0.01
Ashes (g/100 ml)	0.36 ± 0.01	0.35 ± 0.01
Lipids (g/100 ml)	0.17 ± 0.01	0.16 ± 0.01
Protein (g/100 ml)	0.70 ± 0.01	0.59 ± 0.00
Total carbohydrates (g/100 ml)	11.41	10.70
Caloric energetic content (kcal/100 ml)	50	47

**Figure 1:**
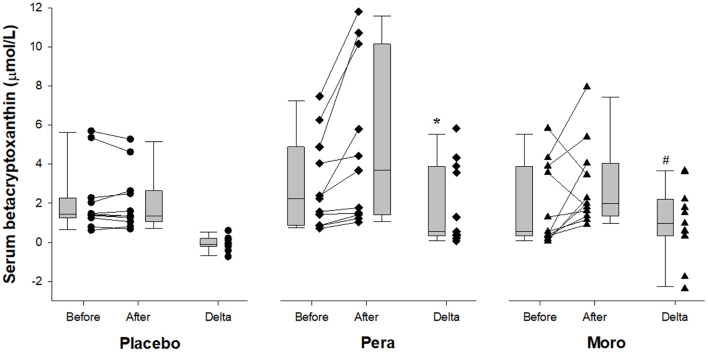
Comparison of serum beta-cryptoxanthin between Placebo, “Pera,” and “Moro” groups (*n* = 11 patients per group). The delta values of the three groups were compared using the Kruskal-Wallis test with Tukey's *post-hoc* test (*p* = 0.005). ^#^Difference between “Moro” and Placebo groups; *difference between “Pera” and Placebo groups.

**Figure 2:**
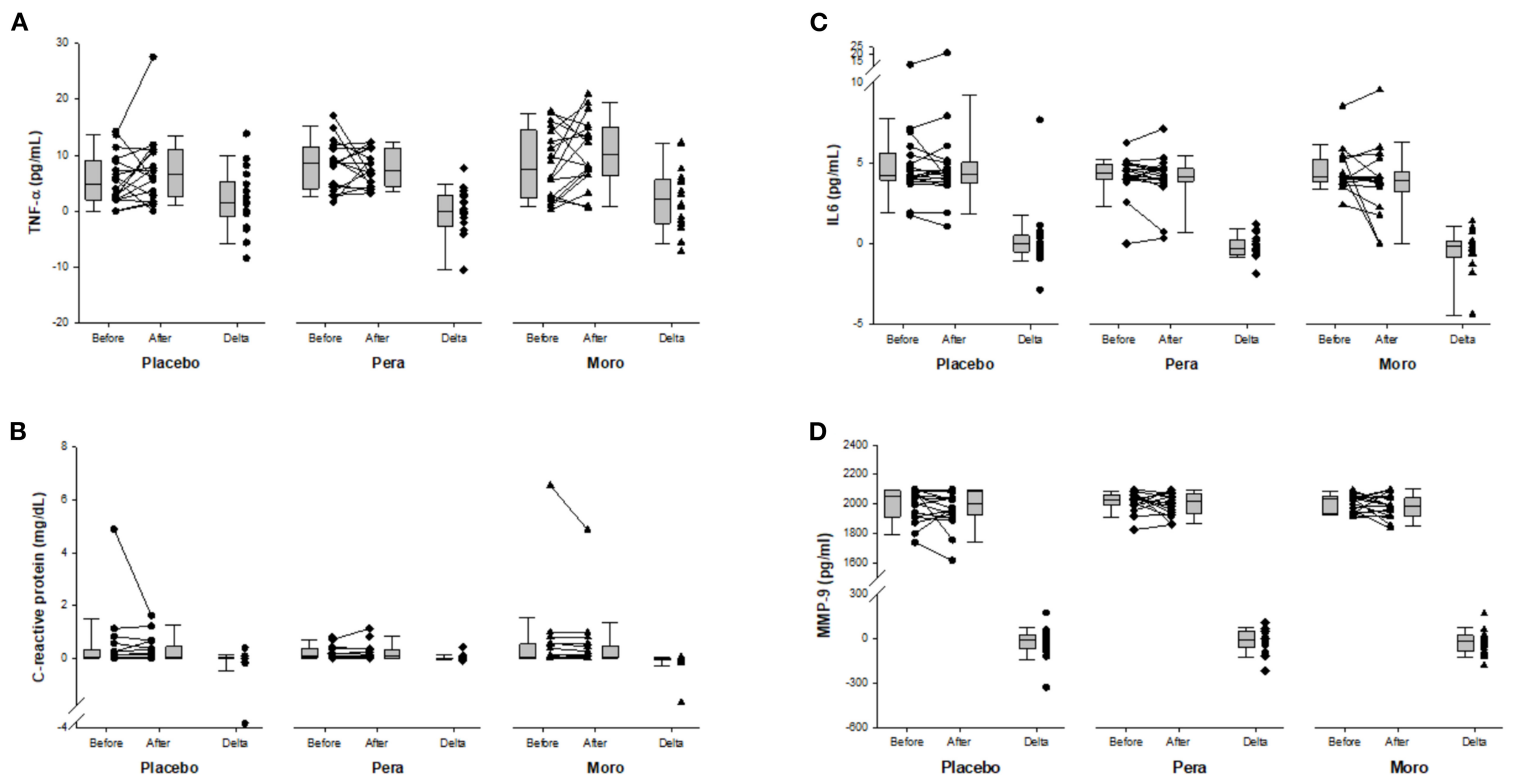
**(A)** Comparison of serum TNF-α between Placebo, “Pera,” and “Moro” groups (*n* = 18 patients per group). The delta values of the three groups were compared using the Kruskal-Wallis test (*p* = 0.250). **(B)** Comparison of serum C-reactive protein between Placebo, “Pera,” and “Moro” groups. The delta values of the three groups were compared using the Kruskal-Wallis test (*p* = 0.158). **(C)** Comparison of serum IL-6 between Placebo, “Pera,” and “Moro” groups. The delta values of the three groups were compared using the Kruskal-Wallis test (*p* = 0.750). **(D)** Comparison of serum MMP-9 between Placebo, “Pera,” and “Moro” groups. The delta values of the three groups were compared using the Kruskal-Wallis test (*p* = 0.843).

As expected, serum values of beta-cryptoxanthin were higher after “Pera” and “Moro” orange juice ingestion (Figure 1). Serum levels of inflammatory biomarkers after beverage ingestion are shown in Figure 2. Serum levels of tumor necrosis factor alpha (TNF-α), IL-6, metalloproteinases (MMP)-9, and CRP did not change after water or juice intake.

**Figure 3:**
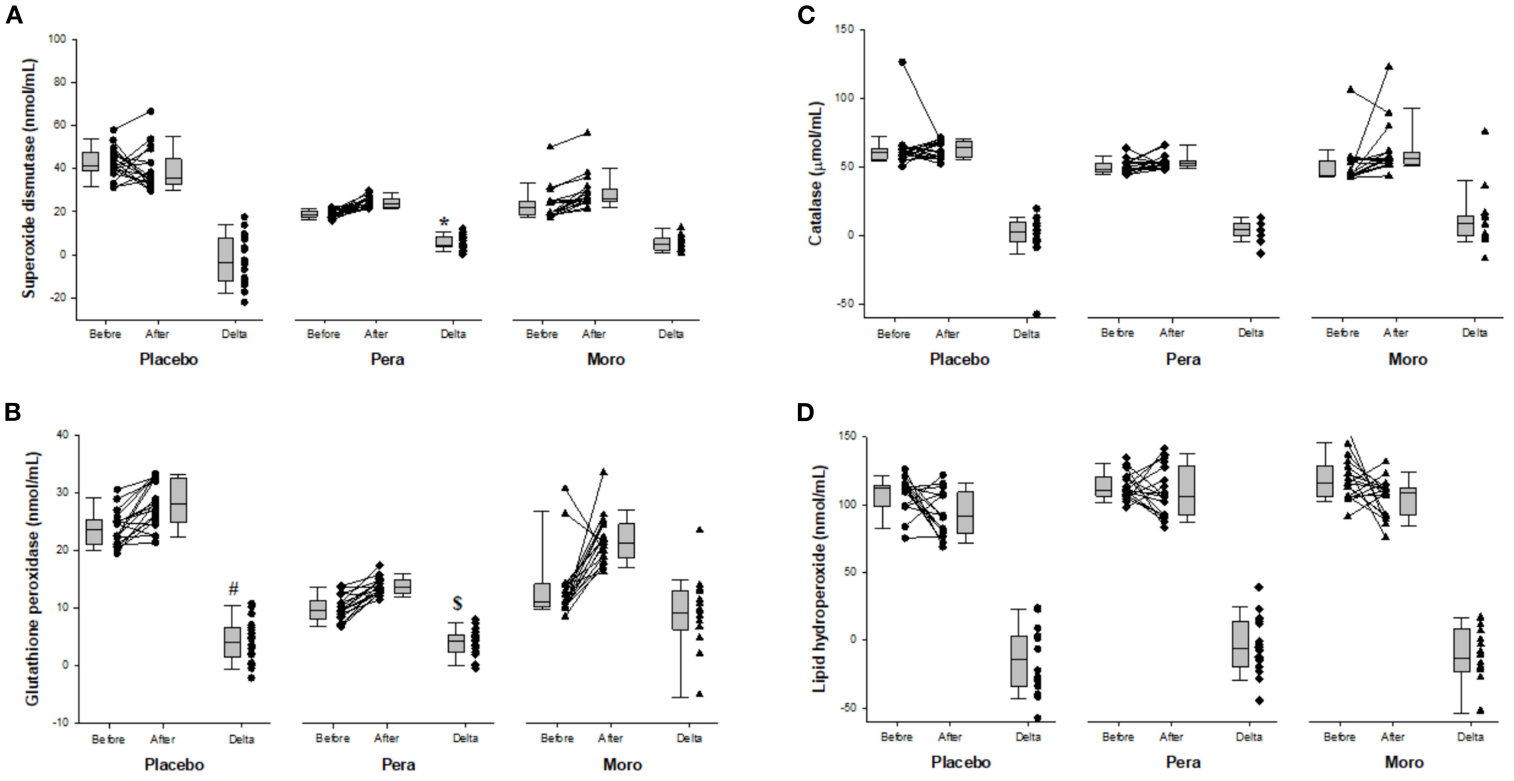
**(A)** Comparison of serum superoxide dismutase between Placebo, “Pera,” and “Moro” groups (*n* = 18 patients per group). The delta values of the three groups were compared using the Kruskal-Wallis test with Tukey's *post-hoc* test (*p* < 0.025). *Difference between “Pera” and Placebo groups. **(B)** Comparison of serum glutathione peroxidase between Placebo, “Pera,” and “Moro” groups. The delta values of the three groups were compared using the Kruskal-Wallis test with Tukey's *post-hoc* test (*p* = 0.004). ^#^Difference between “Moro” and Placebo groups; ^$^difference between “Moro” and “Pera” groups. **(C)** Comparison of serum catalase between Placebo, “Pera,” and “Moro” groups. The delta values of the three groups were compared using the Kruskal-Wallis test (*p* = 0.129). **(D)** Comparison of serum lipid hydroperoxide between Placebo, “Pera,” and “Moro” groups. The delta values of the three groups were compared using the Kruskal-Wallis test (*p* = 0.313).

Figure 3 shows the activity of antioxidant enzymes and of lipid hydroperoxide, a lipid biomarker of oxidative stress. After “Pera” orange juice ingestion, there was an increase in serum activity of superoxide dismutase compared with the placebo group. In addition, there was an increase in serum activity of GPx after “Moro” orange juice ingestion, compared with placebo and “Pera” orange juice ingestion. However, there was no change in lipid hydroperoxide levels.

Regarding circulating miRNAs, we detected increased expression of miR-150-5p, miR-25-3p, and miR-451a in placebo group. In fact, the alterations due to cigarette smoking itself, were responsible for the increased miRNA expression. In addition, these miRNAs were attenuated by orange juices intake (Table 3).

**Table 3:** Circulating miRNAs that are differently expressed after beverage intake in the three groups.

**microRNA expression**	**Fold change**	***P*-value**
**Placebo group**		
hsa-miR-150-5p	6.37	0.023
hsa-miR-25-3p	7.21	0.028
hsa-miR-451a	10.62	0.049
**Pera group**		
No change	–	–
**Moro group**		
No change	–	–

**Figure 4:**
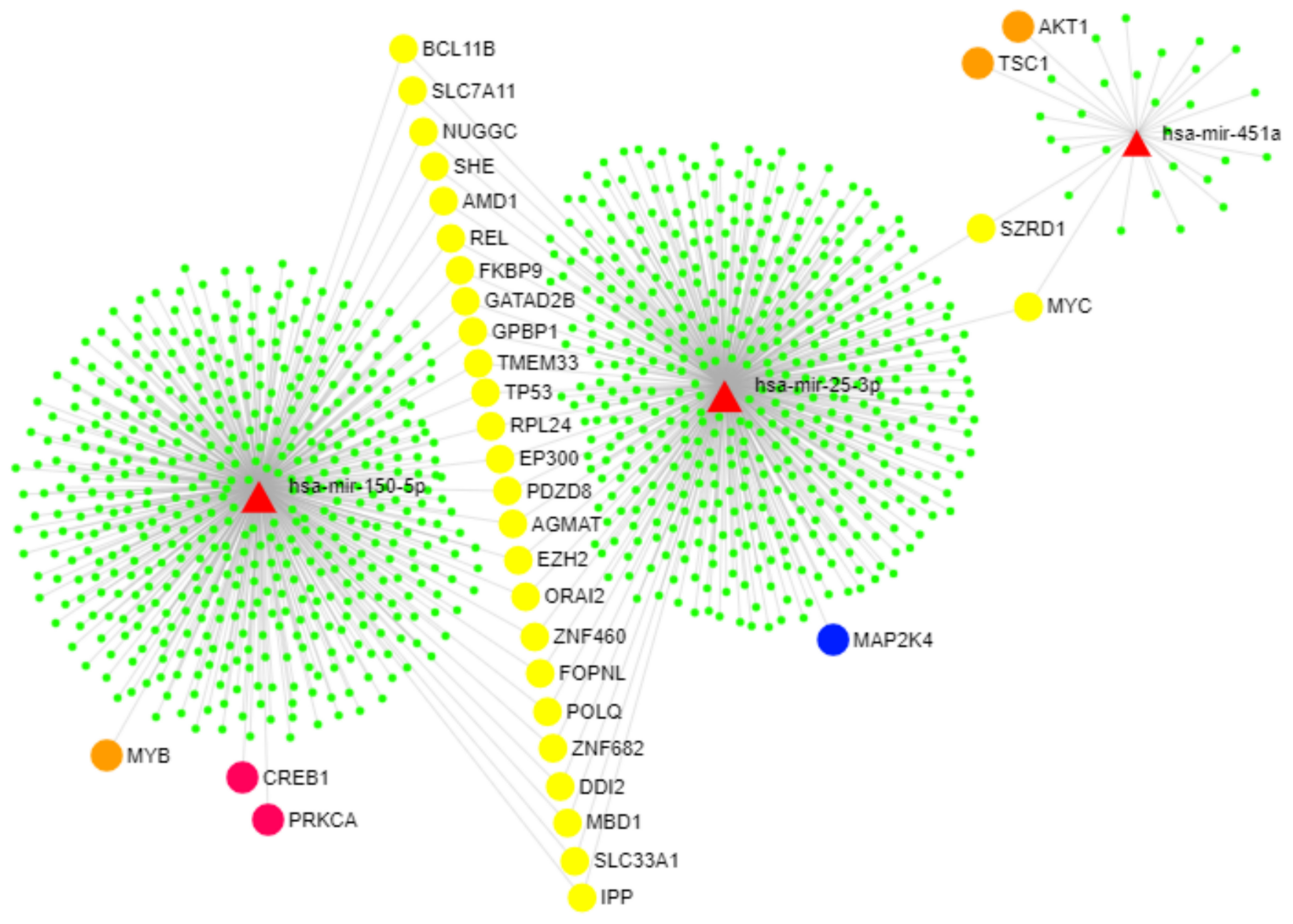
miRNA-target gene network. Genes that modulate the PI3K-AKT and MAPK pathways are highlighted. This figure was generated using the miRNet 2.0 tool (45). The red triangles represent the three upregulated miRNAs (miR-150-5p, miR-25-3p, and miR-451a). The circles represent genes targeted by the identified miRNAs as follows: Orange circles represent genes in the PI3K-AKT pathway, the blue circle represents one gene included in the MAPK pathway, pink circles show genes that participate in both PI3K-AKT and MAPK pathways, yellow circles show genes that are commonly targeted by all of the identified miRNAs, and green circles show other miRNA target genes that participate in other pathways.

**Figure 5:**
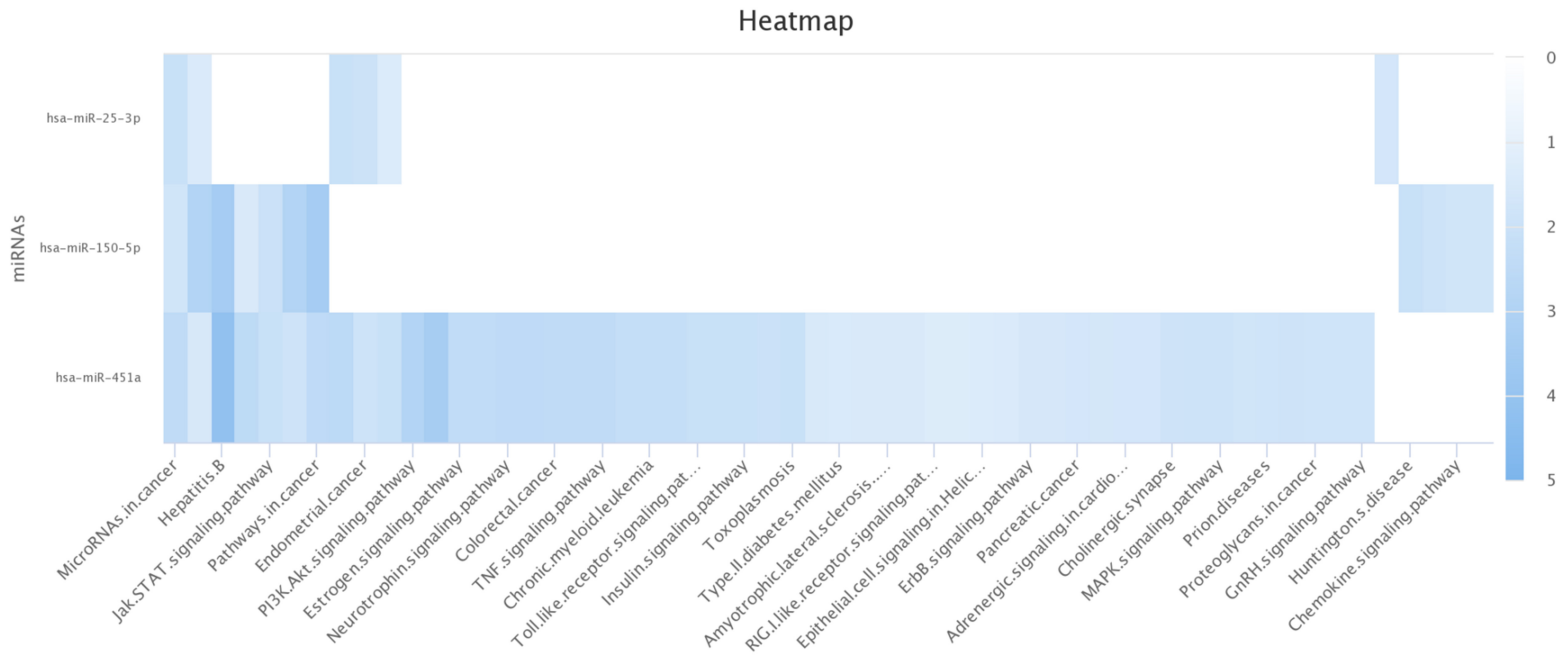
Significantly enriched pathways for the miRNAs. miR-451a is statistically significant for both pathways: PI3K-AKT and MAPK. The other pathways are modulated by the three identified miRNAs (miR-150-3p, mir-25-3p, and mir-451a). This image was generated using the miRPathDB 2.0 tool (46).

Computational analyses using miRDIP showed 244 predicted miRNA targets, regulated for at least two of the identified miRNAs (Supplementary Table 1). Pathway enrichment analysis (Enrichr tool) identified statistically significantly enriched pathways including miRNA-target genes according to KEGG, BioCarta, Panther, and BioPlanet databases (Supplementary Table 2). Figure 4 shows miRNA-target gene interaction networks. Notably, oxidative stress, phosphatidylinositol 3-kinase (PI3K)-Akt, and mitogen-activated protein kinase (MAPK) signaling pathways were among the enriched pathways regulated by genes targeted by miRNAs (Figure 5).

## Discussion

To the best of our knowledge, this is the first study to evaluate global miRNA expression profiles after acute intake of orange juice in healthy smokers. Here, we used the NanoString nCounter® technology, a probe-based assay that avoids the bias introduced by amplification in PCR-based assays, which has high sensitivity and specificity, key features for diagnostic applications (54, 55). Notably, consumption of both types of orange juice induced the same response with decreased plasma expression levels of a subset of miR-150-5p, miR-25-3p, and miR-451a. It is important to observe that serum beta-cryptoxanthin levels increased after orange juice intake, confirming that the juice was absorbed.

The role of identified miRNAs and their target genes in oxidative stress, PI3K-Akt, and MAPK signaling pathways suggests a putative mechanism associated with the health-beneficial effects of orange juice intake. Importantly, such pathways have been previously shown in clinical and experimental studies assessing supplementation with the citrus flavonoid hesperidin. Such studies demonstrated the effects of orange juice or hesperidin on oxidative stress, mainly through the Nrf2 pathway (56–60). Nrf2 is a leucine zipper transcriptional factor that regulates transcription of several antioxidant enzymes such as SOD, CAT, and GPx. Its antioxidant properties were demonstrated in experimental models of hepatocarcinogenesis, in the hearts of senescent rats, in healthy subjects, and in clinical trials, improving aerobic performance (24, 56–58).

Here, “Pera” orange juice only increased the activity of SOD, and “Moro” orange juice only increased the activity of GPx. As indicated by their blood-colored flesh, “Moro” oranges have larger amounts of anthocyanins than do “Pera” oranges (19, 20). Anthocyanins are able to activate the expression of glutathione-related enzymes, through Nrf2 activation, which could explain the greater effect of Moro orange juice in improving the activity of glutathione peroxidase observed in our study (61). However, the interventions were not sufficient to decrease lipid hydroperoxide levels. These results may be explained by the assessment of an acute effect of orange juice intake.

The MAPKs comprise a family of protein kinases related to cell growth, inflammation, and oxidative cell damage (59). Three major classes of MAPKs are extracellular-signal regulated kinases 1 and 2 (ERK1/2), p38 MAPK/stress-activated protein kinases, and c-Jun N-terminal kinases (JNKs). Hesperidin supplementation was shown to modulate the MAPK pathway in experimental models of vascular and neuroinflammation, which confirms our findings (59, 60).

Experimental studies on cancer, myocardial ischemia/reperfusion injury, and arthritis have already shown the effects of hesperidin in modulation of the PI3K-Akt pathway (62–65). The PI3K/Akt signaling pathway regulates various cellular responses such as cell growth, proliferation, survival, metabolism, and inflammation. The PI3Ks are a family of lipid kinases that phosphorylate the 3′-OH group of the inositol ring of phosphatidylinositols on the plasma membrane (62). These enzymes are activated by either upstream tyrosine kinase receptors or G protein-coupled receptors and mediate multiple cellular processes (62). PI3K/Akt signaling is essential for regulation of inflammatory responses, at least in part via controlling intracellular NF-κB activity (63). Our data did not show NF-κB associated with orange juice intake. Furthermore, TNF-α, IL-6, MMP-9, and CRP serum levels were not reduced following orange juice consumption. This lack of effect on inflammatory biomarkers is likely due to the low grade of inflammation detected in our study participants (healthy smokers). In addition, altered expression of miRNA is supposed to occur earlier than protein alteration.

Although we did not observe an improvement in the levels of classical inflammatory or oxidative stress biomarkers, in active smokers, we were able to show that orange juice intake modulates the expression levels of a specific subset of circulating plasma miRNAs. Our study results shed light on important pathways that could mediate the beneficial effects of orange juice consumption, associated with miRNA modulation.

Among the limitations of our study, we included individuals from a single medical center and evaluated only one acute ingestion of orange juice. Despite these limitations, this is the first study to evaluate global miRNA expression in plasma from healthy smokers after orange juice intake, using a highly sensitive, specific, and reproducible probe-based assay.

Finally, future studies could focus on the evaluation of the genes involved in the identified molecular pathways. In addition, its relationship with the inflammatory process and oxidative stress due to chronic orange juice intake could be assessed.

In conclusion, tobacco smokers showed an increase in the expression of a specific subset of miRNAs (miR-150-5p, miR-25-3p, and miR-451a), attenuated by orange juice intake. miRNA target genes regulate enriched pathways of oxidative stress, PI3K-Akt, and MAPK signaling, which are commonly associated with smoking-related diseases.

## Data Availability Statement

The original contributions presented in the study are included in the article/Supplementary Material, further inquiries can be directed to the corresponding author/s.

## Ethics Statement

The study was approved by the Botucatu Medical School Ethics Committee (85655718.1.0000.5411). The patients/participants provided their written informed consent to participate in this study.

## Author Contributions

MM, MD, LZ, SP, and PR designed research. MD, EB, MC, AS, FC-M, TF, CC, AF, PSA, and BP conducted research, provided essential reagents, or provided essential materials. ST, PR, MD, MM, MR, and LZ analyzed data or performed statistical analysis. MM, MD, and PR wrote paper. MD and MM had primary responsibility for final content. All authors read and approved the final manuscript.

## Funding

This study was funded by FAPESP (Proc. n.2017/23523-0 and n.2016/20646-0), the Botucatu Medical School University Hospital, São Paulo State University (UNESP), Botucatu, Brazil, and CAPES.

## Conflict of Interest

The authors declare that the research was conducted in the absence of any commercial or financial relationships that could be construed as a potential conflict of interest.

## Publisher's Note

All claims expressed in this article are solely those of the authors and do not necessarily represent those of their affiliated organizations, or those of the publisher, the editors and the reviewers. Any product that may be evaluated in this article, or claim that may be made by its manufacturer, is not guaranteed or endorsed by the publisher.
