# Supplementary material for: Orange Juice Attenuates Circulating miR-150-5p, miR-25-3p, and miR-451a in Healthy Smokers: A Randomized Crossover Study
Source: Front Nutr. 2021 Dec 24;8:775515. doi: 10.3389/fnut.2021.775515 (PMC8740272; doi:10.3389/fnut.2021.775515)
Supplement: Supplementary file 1 [file Data_Sheet_1.PDF]

Supplementary Table 1: 244 predicted miRNA targets, regulated for at least two of the identified miRNAs (Computa-tional analyses using miRDIP).

Generated at: 2020/09/28 16:41:48
mirDIP version: 4.1.11.1.
Database version: 4.1.0.3

Micro RNAs: hsa-miR-150-5p
hsa-miR-25-3p
hsa-miR-451a

Minimum Score class - Very High

Results (244)

Gene	Uniprot \ miRNAs	hsa-miR-150-5p	hsa-miR-25-3p	hsa-miR-451a	Shared miRNAs
AARS	P49588	Y	Y		2
ABI2	Q9NYB9	Y	Y		2
ACADL	P28330		Y	Y	2
ACOX1	Q15067	Y	Y		2
ADAM10	O14672		Y	Y	2
ADAM19	Q9H013	Y	Y		2
ADAMTS5	Q9UNA0	Y	Y		2
ADARB1	P78563	Y		Y	2
ADD1	P35611	Y	Y		2
AEBP2	Q6ZN18	Y		Y	2
AFF1	P51825	Y	Y		2
AFF4	Q9UHB7	Y	Y		2
AGO04	Q9HCK5	Y	Y		2
AHCTF1	Q8WYP5	Y	Y		2
AIFM2	Q9BRQ8	Y	Y		2
AKAP6	Q13023	Y	Y		2
ANKRD12	Q6UB98	Y	Y		2

AP1G1	O43747	Y	Y		2
ARF1	P84077	Y	Y		2
ATG14	Q6ZNE5	Y	Y		2
ATXN1	P54253	Y	Y		2
AZIN1	O14977	Y	Y		2
BAZ2A	Q9UIF9	Y	Y		2
BCAT1	P54687	Y	Y		2
BDNF	P23560	Y	Y		2
BMPR2	Q13873	Y	Y		2
BSDC1	Q9NW68	Y	Y		2
BSN	Q9UPA5	Y	Y		2
BTBD9	Q96Q07	Y		Y	2
BTLA	Q7Z6A9	Y	Y		2
C19orf12	Q9NSK7	Y	Y		2
C1orf21	Q9H246	Y	Y		2
C21orf91	Q9NYK6		Y	Y	2
CACNA1C	Q13936	Y	Y		2
CACNA2D1	P54289	Y	Y		2
CCNJ	Q5T5M9	Y	Y		2
CD44	P16070	Y	Y		2
CELF2	O95319	Y	Y		2
CERK	Q8TCT0		Y	Y	2
CHD2	O14647	Y	Y		2
CMTM6	Q9NX76	Y		Y	2
CNOT6L	Q96LI5	Y	Y		2
COL19A1	Q14993	Y	Y		2
CPD	O75976	Y		Y	2
CPEB4	Q17RY0	Y	Y		2
CREB1	P16220	Y	Y		2
CREB3L2	Q70SY1	Y	Y		2
CTNNBIP1	Q9NSA3		Y	Y	2
CXADR	P78310	Y	Y		2
CYLD	Q9NQC7	Y	Y	Y	3

DBT	P11182	Y	Y		2
DCAF6	Q58WW2	Y	Y		2
DCAF8	Q5TAQ9	Y	Y		2
DCP2	Q8IU60	Y	Y		2
DCX	O43602	Y	Y		2
DDX3Y	O15523	Y	Y		2
DENND5B	Q6ZUT9	Y	Y		2
DLGAP2	Q9P1A6	Y	Y		2
DPP10	Q8N608	Y	Y		2
DSTYK	Q6XUX3	Y	Y		2
DUSP10	Q9Y6W6	Y	Y		2
DYRK1A	Q13627	Y	Y		2
E2F3	O00716	Y	Y		2
EGR1	P18146	Y	Y		2
EGR2	P11161	Y	Y		2
EIF2AK3	Q9NZJ5	Y		Y	2
EIF5	P55010	Y	Y		2
EPG5	Q9HCE0	Y	Y		2
EREG	O14944	Y	Y	Y	3
FAM117B	Q6P1L5	Y	Y		2
FAM133B	Q5BKY9	Y	Y		2
FAM91A1	Q658Y4		Y	Y	2
FBXO33	Q7Z6M2		Y	Y	2
FBXW11	Q9UKB1	Y	Y		2
FKBP14	Q9NWM8	Y	Y		2
FOPNL	Q96NB1	Y	Y		2
FOSL2	P15408	Y	Y		2
FOXP1	Q9H334	Y	Y		2
FREM2	Q5SZK8	Y	Y		2
FZD4	Q9ULV1	Y	Y		2
GAA	P10253	Y	Y		2
GAN	Q9H2C0	Y	Y		2
GATAD2B	Q8WXI9	Y	Y	Y	3

GDI1	P31150	Y		Y	2
GID4	Q8IVV7	Y	Y		2
GIGYF2	Q6Y7W6	Y	Y		2
GLCE	O94923	Y	Y		2
GLRA1	P23415	Y	Y		2
GLYR1	Q49A26	Y	Y		2
GOLGA3	Q08378	Y	Y		2
GOLGA7	Q7Z5G4	Y	Y		2
GPATCH8	Q9UKJ3	Y	Y		2
GPR85	P60893	Y	Y		2
HECTD1	Q9ULT8	Y	Y		2
HEG1	Q9ULI3	Y	Y		2
HMGA2	P52926	Y	Y		2
HNRNPA3	P51991	Y	Y		2
HNRNPU	Q00839	Y	Y		2
IKZF2	Q9UKS7	Y	Y		2
INSR	P06213	Y	Y		2
IPO5	O00410	Y	Y		2
ITCH	Q96J02	Y	Y		2
ITGA6	P23229	Y	Y		2
ITGAV	P06756	Y	Y		2
ITGB3	P05106	Y	Y		2
ITPRIPL2	Q3MIP1	Y	Y		2
ITSN1	Q15811	Y	Y		2
JADE1	Q6IE81	Y	Y		2
JARID2	Q92833	Y	Y		2
JPH2	Q9BR39	Y	Y		2
KAT6A	Q92794	Y	Y		2
KCNJ3	P48549	Y	Y		2
KIF1B	O60333	Y	Y		2
KIF3B	O15066	Y	Y		2
KLF12	Q9Y4X4	Y	Y		2
KLHDC10	Q6PID8	Y	Y		2

KLHL15	Q96M94	Y	Y		2
KLHL3	Q9UH77	Y	Y		2
KMT5B	Q4FZB7	Y	Y		2
KSR2	Q6VAB6	Y	Y		2
LCOR	Q96JN0	Y	Y		2
LPP	Q93052	Y	Y		2
LRRC8B	Q6P9F7	Y	Y		2
MAFK	O60675	Y	Y		2
MAP1B	P46821	Y	Y		2
MAP2K4	P45985	Y	Y		2
MAP4K4	O95819	Y	Y		2
MBNL3	Q9NUK0	Y	Y		2
MEF2D	Q14814		Y	Y	2
METAP1	P53582	Y	Y		2
MMD	Q15546	Y	Y		2
MMP16	P51512	Y	Y		2
MRPS25	P82663	Y	Y		2
MTMR9	Q96QG7	Y	Y		2
MYB	P10242	Y	Y		2
MYO1B	O43795	Y	Y		2
MYO5A	Q9Y4I1	Y	Y		2
NEFM	P07197	Y	Y		2
NEGR1	Q7Z3B1	Y	Y		2
NEMP1	O14524	Y	Y		2
NFAT5	O94916	Y	Y		2
NFATC2IP	Q8NCF5	Y	Y		2
NKX2-4	Q9H2Z4	Y	Y		2
NLK	Q9UBE8	Y	Y		2
NPTN	Q9Y639		Y	Y	2
NSMAF	Q92636		Y	Y	2
NTRK2	Q16620	Y	Y		2
NXPE3	Q969Y0	Y	Y		2
P3H3	Q8IVL6	Y	Y		2

PAFAH1B1	P43034	Y	Y		2
PAG1	Q9NWQ8	Y	Y		2
PAPD5	Q8NDF8	Y	Y		2
PAPD7	Q5XG87	Y	Y		2
PAX5	Q02548	Y	Y		2
PDS5B	Q9NTI5	Y	Y		2
PDXDC1	Q6P996	Y	Y		2
PFN2	P35080	Y	Y		2
PGM2L1	Q6PCE3	Y	Y		2
PHC3	Q8NDX5	Y	Y		2
PHF3	Q92576	Y	Y		2
PHTF2	Q8N3S3	Y	Y		2
PI15	O43692	Y	Y		2
PIK3AP1	Q6ZUJ8	Y	Y		2
PIK3R3	Q92569	Y	Y		2
PITPNA	Q00169	Y	Y		2
PLEKHA1	Q9HB21	Y	Y		2
PLEKHA6	Q9Y2H5	Y	Y		2
PPM1B	O75688	Y	Y		2
PPP1R3A	Q16821	Y	Y		2
PPP1R9A	Q9ULJ8	Y	Y		2
PRICKLE2	Q7Z3G6	Y	Y	Y	3
PRKCA	P17252	Y	Y		2
PSD3	Q9NYI0	Y	Y		2
PTGFR	P43088	Y	Y		2
PTPRJ	Q12913	Y	Y		2
PWWP2A	Q96N64	Y	Y		2
RAB8B	Q92930	Y	Y		2
RANBP9	Q96S59	Y	Y		2
RBFOX1	Q9NWB1	Y	Y		2
RGS3	P49796	Y	Y		2
RIMBP2	O15034	Y	Y		2
RIMS2	Q9UQ26	Y	Y		2

RNF141	Q8WVD5	Y	Y		2
RPRD2	Q5VT52	Y	Y		2
SAMD4B	Q5PRF9	Y		Y	2
SCD5	Q86SK9	Y	Y		2
SDC2	P34741	Y	Y		2
SEC31B	Q9NQW1	Y	Y		2
SEL1L	Q9UBV2	Y	Y		2
SEMA3A	Q14563	Y	Y		2
SEMA6D	Q8NFY4	Y	Y		2
SETD5	Q9C0A6	Y	Y	Y	3
SLC12A5	Q9H2X9	Y	Y		2
SLC1A2	P43004	Y	Y		2
SLC2A3	P11169	Y	Y		2
SLC32A1	Q9H598	Y	Y		2
SLC4A4	Q9Y6R1	Y	Y		2
SLC6A17	Q9H1V8	Y	Y		2
SLC7A11	Q9UPY5	Y	Y		2
SMU1	Q2TAY7	Y	Y		2
SNX13	Q9Y5W8	Y	Y		2
SNX30	Q5VWJ9	Y	Y		2
SORCS3	Q9UPU3	Y	Y		2
SOX11	P35716	Y	Y		2
SP1	P08047	Y	Y		2
SREK1	Q8WXA9	Y	Y	Y	3
SREK1IP1	Q8N9Q2	Y	Y		2
STK39	Q9UEW8	Y	Y		2
SYPL1	Q16563	Y	Y		2
SZRD1	Q7Z422	Y		Y	2
TEAD1	P28347	Y	Y		2
TEK	Q02763	Y	Y		2
TEX2	Q8IWB9	Y	Y		2
TGFBR1	P36897	Y	Y		2
TMCC1	O94876	Y	Y		2

TMOD1	P28289	Y	Y		2
TMSB4Y	O14604	Y	Y		2
TNRC6B	Q9UPQ9	Y	Y		2
TRIM66	O15016	Y		Y	2
TSC1	Q92574		Y	Y	2
TTPAL	Q9BTX7	Y	Y		2
UACA	Q9BZF9	Y	Y		2
UBE2Z	Q9H832	Y	Y		2
UBR1	Q8IWV7	Y	Y		2
UST	Q9Y2C2	Y	Y		2
VAPA	Q9P0L0	Y		Y	2
VEZF1	Q14119	Y	Y		2
VSIG10	Q8N0Z9	Y	Y		2
WDFY3	Q8IZQ1	Y	Y		2
WDR37	Q9Y2I8	Y	Y		2
XPNPEP3	Q9NQH7	Y	Y		2
XYLT2	Q9H1B5	Y	Y		2
YTHDF3	Q7Z739		Y	Y	2
YWHAZ	P63104		Y	Y	2
ZDHHC2	Q9UIJ5	Y	Y		2
ZFP91	Q96JP5	Y	Y		2
ZFYVE21	Q9BQ24	Y	Y		2
ZNF148	Q9UQR1	Y	Y		2
ZNF189	O75820	Y	Y		2
ZNF25	P17030	Y	Y		2
ZNF331	Q9NQX6	Y	Y		2
ZNF365	Q70YC4	Y	Y		2
ZNF654	Q8IZM8		Y	Y	2
ZNF711	Q9Y462	Y	Y		2

Supplementary Table 2: Pathway enrichment analysis (Enrichr tool) identified statistically significantly enriched pathways including miRNA-target genes according to KEGG, BioCarta, Panther, and BioPlanet databases

**Molecular Pathways**	**Target genes**	**P value**	**Combined score**	**Data bank**
PI3K-Akt signaling pathway	NTRK2, BDNF, INSR, ITGB3, PIK3R3, PRKCA, TSC1, YWHAZ, EREG, CREB1, MYB, CREB3L2, ITGA6, ITGAV, TEK, PIK3AP1	<0.001	43.55	KEGG 2019
Parathyroid hormone synthesis, secretion and action	EGR1, CREB1, MMP16, SP1, CREB3L2, PRKCA, MEF2D	<0.001	43.51	KEGG 2019
MAPK signaling pathway	MAP2K4, NTRK2, BDNF, CACNA2D1, INSR, PRKCA, CACNA1C, NLK, TGFBR1, EREG, PPM1B, DUSP10, TEK, MAP4k4	<0.001	42.72	KEGG 2019
Hepatitis B	MAP2K4, EGR2, CREB1, CREB3L2, PIK3R3, PRKCA, E2F3, YWHAZ, TGFBR1	<0.001	38.99	KEGG 2019
Arrhythmogenic right ventricular cardiomyopathy (ARVC)	ITGB3, CACNA2D1, ITGAV, ITGA6, CACNA1C	0.0018	35.77	KEGG 2019
Oxidative Stress Induced Gene Expression Via Nrf2 Homo sapiens h arenrf2Pathway	CREB1, PRKCA, MAFK	0.0013	91.02	BioCarta 2016
Regulation of eIF2 Homo sapiens h eif2Pathway	EIF5, EIF2AK3	0.0076	72.76	BioCarta 2016
Effects of calcineurin in Keratinocyte Differentiation Homo sapiens h calcineurinPathway	SP1, PRKCA	0.0106	57.36	BioCarta 2016

MAPKinase Signaling Pathway Homo sapiens h mapkPathway	MAP2K4, CREB1, SP1, MEF2D, MAP4k4	<0.001	54.33	BioCarta 2016
Role of Erk5 in Neuronal Survival Homo sapiens h erk5Pathway	CREB1, BANF, MEF2D	0.0038	52.73	BioCarta 2016
p38 MAPK pathway Homo sapiens P05918	MAP2K4, DUSP10, MEF2D	0.0069	38.29	Panther 2016
Insulin/IGF pathway-protein kinase B signaling cascade Homo sapiens P00033	INRS, PIK3R3, TSC1	0.0081	34.81	Panther 2016
PI3 kinase pathway Homo sapiens P00048	INRS, PIK3R3, YWHAZ	0.0145	24.78	Panther 2016
Oxidative stress response Homo sapiens P00046	MAP2K4, DUSP10	0.0343	23.04	Panther 2016
Alzheimer disease-amyloid secretase pathway Homo sapiens P00003	ADAM10, PRKCA, CACNA1C	0.0309	15.26	Panther 2016
Post-transcriptional silencing by small RNAs	AGO4, TNRC6B	0.0029	136.13	BioPlanet 2019
eIF2 regulation	EIF5, EIF2AK3	0.0076	72.76	BioPlanet 2019
Oxidative stress-induced gene expression via Nrf2	CREB1, PRKCA, MAFK	0.0020	72.61	BioPlanet 2019
Platelet endothelial cell adhesion molecule 1 (PECAM1) interactions	ITGB3, ITGAV	0.0090	64.31	BioPlanet 2019
SREBP signaling	CREB1, SP1, SEC31B	0.0030	59.51	BioPlanet 2019
